# Vns as alternative treatment for maintenance ect in a patient with treatment resistant depression - a case study

**DOI:** 10.1192/j.eurpsy.2021.1308

**Published:** 2021-08-13

**Authors:** C. Migchels, P. Sienaert

**Affiliations:** University Psychiatric Centre, KULeuven, Kortenberg, Belgium

**Keywords:** VNS, ECT, Treatment Resistant Depression, Case study

## Abstract

**Introduction:**

Vagus Nerve Stimulation (VNS) is a neuromodulatory intervention which involves attaching an electrode to the vagus nerve. Studies investigating VNS as an acute treatment method for treatment resistent depression have shown very limited results, however there are data suggesting that VNS might have a beneficial effect on a longer term. There are also studies that suggest that a history of response to ECT might indicate a higher response rate to VNS. VNS was suggested as treatment for a patient who received maintenance ECT for treatment resistant unipolar depression during 9 years. 3 months after VNS was implanted, ECT was stopped due to the Covid-19 pandemic. In this case study we will review the patient’s response to treatment with VNS and the sudden stop of long-term ECT treatment.

**Objectives:**

To review the response to acute and maintenance ECT and VNS in this patient diagnosed with treatment resistant unipolar depression, and to compare this to the data suggesting VNS as an alternative treatment method for maintenance ECT in patients with treatment resistant depression.

**Methods:**

Using the extensive data collected during the patient’s treatment, we will review the clinical response and side-effect burden of this patient to acute and maintenance ECT and to VNS.

**Results:**

The patient showed a vast improvement in depressive symptoms a few months after start of VNS treatment, while long-term maintenance ECT was stopped.

**Conclusions:**

This patient’s response to VNS supports the data suggesting VNS as an alternative treatment method for maintenance ECT in patients with treatment resistant depression.

**Conflict of interest:**

This patient received VNS treatment as part of a study conducted in our centre (UPC KULeuven) with support of Livanova. Me nor my supervisor (prof. Sienaert Pascal) are directly involved in this study. I have received no financial or other compensation fr

